# Sleep Habits and Maternal Anxiety in Children with Growing Pains: A Comparative Cross-Sectional Study

**DOI:** 10.3390/children13101429

**Published:** 2026-10-18

**Authors:** Sema Nur Taşkın, Tuğba Karaca Ahat, Sevil Aydoğan Gedik, Hande Ayan Karanfil, Şule Pektaş Leblebicier, Eda Boz Öncel, Tuğçe Arman Mut, Fatma Özbek Tezcan

**Affiliations:** 1 Division of Pediatric Rheumatology, Department of Pediatrics, Eskişehir City Hospital, Eskişehir 26080, Türkiye; 2 Division of Developmental Pediatrics, Department of Pediatrics, Eskişehir City Hospital, Eskişehir 26080, Türkiye; 3 Department of Public Health, Eskişehir Provincial Directorate of Health, Eskişehir 26020, Türkiye; 4 Department of Pediatrics, Eskişehir City Hospital, Eskişehir 26080, Türkiye; 5 Division of Pediatric Nephrology, Department of Pediatrics, Eskişehir City Hospital, Eskişehir 26080, Türkiye

**Keywords:** growing pains, sleep habits, sleep disturbance, children’s sleep habits questionnaire, pain severity, maternal anxiety, pediatric pain

## Abstract

**Highlights:**

**What are the main findings?**

- Children with growing pains had significantly greater sleep disturbance than healthy controls.
- Greater pain severity was independently associated with sleep disturbance in children with growing pains.

**What is the implication of the main finding?**

- Sleep assessment should be considered in children with growing pains, particularly those reporting more severe pain.
- Maternal anxiety may contribute to sleep-related difficulties, although its independent association was not statistically significant within the growing-pains subgroup.

**Abstract:**

**Background:** Growing pains commonly present as recurrent evening or nocturnal musculoskeletal pain in childhood. Evidence on associated sleep disturbance and maternal anxiety is limited. We compared affected children with healthy controls and examined sleep-related factors. **Methods:** A total of 138 children aged 4–12 years were included in this single-center study, comprising 71 children with growing pains and 67 age- and sex-comparable healthy controls. Growing pains were diagnosed using the Peterson criteria. Pain severity was assessed using age-appropriate self-report instruments, while sleep habits and maternal anxiety were evaluated with the Children’s Sleep Habits Questionnaire (CSHQ) and Generalized Anxiety Disorder-7 (GAD-7), respectively. Multivariable regression identified factors associated with CSHQ total scores. **Results:** Children with growing pains had higher CSHQ total scores than healthy controls [49.0 (33.0–81.0) vs. 42.0 (31.0–79.0), *p* < 0.001], and scores above the screening cut-off were more frequent (76.1% vs. 52.2%, *p* = 0.003). Maternal GAD-7 scores did not differ between groups (*p* = 0.531). After multivariable adjustment, growing pains (*p* = 0.020) and higher maternal GAD-7 scores (*p* = 0.003) were independently associated with higher CSHQ scores in the overall sample. Among children with growing pains, greater pain severity was independently associated with higher CSHQ scores (*p* = 0.033), whereas maternal GAD-7 score was not. **Conclusions:** Children with growing pains had greater sleep disturbance than healthy controls after multivariable adjustment. Higher maternal GAD-7 scores were associated with sleep disturbance overall, but not within the growing-pains group. Greater pain severity was independently associated with sleep disturbance in affected children. These findings support sleep assessment in this population.

## 1. Introduction

Growing pains (GP), also referred to as benign nocturnal limb pains of childhood, are a frequent source of musculoskeletal complaints in children. Reported prevalence estimates vary considerably, ranging from 2.6% to 36.9% across different populations and diagnostic approaches [1,2]. GP typically affects children between 3 and 12 years of age and is characterized by intermittent bilateral lower extremity pain, usually occurring during the evening or nighttime and resolving by morning without objective signs of inflammation. Although considered a benign and self-limiting condition, recurrent pain episodes may negatively affect children’s daily activities, sleep, and quality of life [2].

Although the exact pathophysiology of growing pains remains unclear, current evidence suggests that the condition is multifactorial, with proposed mechanisms including lower pain thresholds, reduced bone strength, vitamin D deficiency, hypermobility or increased physical activity. In addition, family history and psychosocial factors may also contribute to disease onset [3].

Sleep disturbances are increasingly recognized in pediatric populations affected by chronic or recurrent pain. Previous studies have suggested a bidirectional relationship between sleep and pain, in which pain may contribute to sleep disturbance, while disturbed sleep may increase pain perception and pain sensitivity. Poor sleep may also contribute to impaired daytime functioning, behavioral problems, and reduced health-related quality of life in children with recurrent pain. Therefore, evaluating sleep habits together with pain severity may provide clinically relevant information for a more comprehensive assessment of children with growing pains [4].

Pain is the principal clinical manifestation of growing pains. Recent evidence has highlighted the importance of using standardized, age-appropriate, valid, and reliable instruments to assess pain characteristics and severity in children with growing pains [5,6].

In addition, psychosocial and family-related factors, particularly parental anxiety and emotional distress, are thought to influence pain perception and pain-related disability in children [7,8]. Parental psychological well-being may be particularly important in childhood pain disorders, as parental anxiety and psychological distress have been associated with greater pain intensity, poorer physical and psychosocial functioning, and altered parental perception and reporting of children’s symptoms [9,10]. Nevertheless, despite increasing interest in psychosocial factors, maternal anxiety has received relatively little attention in studies of growing pains, and evidence regarding sleep habits in this population also remains limited [5].

Overall, studies jointly examining pain severity, sleep habits, and maternal anxiety in children with growing pains remain scarce. Accordingly, this study aimed to compare sleep habits and maternal anxiety between children with growing pains and healthy controls and, among children with growing pains, to examine the associations of pain severity and maternal anxiety with sleep disturbance.

## 2. Materials and Methods

### 2.1. Study Design and Participants

We conducted this single-center, cross-sectional observational study at the Pediatric Rheumatology Outpatient Clinic of Eskişehir City Hospital. Participants were recruited between November 2025 and July 2026. The study focused on evaluating sleep habits, pain severity, and maternal anxiety in children with growing pains.

Ethical approval for the study was obtained from the Scientific Research Ethics Committee of Eskişehir City Hospital (Decision No. ESH/BAEK 2025/241; 8 October 2025). Prior to enrollment, written informed consent was obtained from the parents or legal guardians of all participants.

Children aged 4–12 years who were diagnosed with growing pains according to the Peterson diagnostic criteria were included in the study [11,12]. Typical diagnostic features included intermittent bilateral lower extremity pain occurring predominantly during the evening or nighttime, absence of limitation in physical activity, and normal physical examination findings. Age- and sex-comparable healthy children attending different pediatric outpatient clinics within the same hospital for routine well-child visits during the same study period, with no chronic musculoskeletal disease, chronic pain syndrome, neurological disorder, or inflammatory disease, were considered for the control group. Children who met the eligibility criteria and whose families agreed to participate were included. Recruitment was not conducted using a predefined consecutive sampling protocol. The numbers of potentially eligible control-group children whose families declined participation or who were excluded were not systematically recorded.

Children with chronic inflammatory diseases, neurological disorders, orthopedic disorders, chronic systemic illnesses, developmental delay, psychiatric disorders, known sleep disorders, or active infection were excluded from the study.

As part of the routine clinical evaluation, serum ferritin, 25-hydroxyvitamin D, vitamin B12, and folate levels were measured in all children evaluated for growing pains. These tests were not systematically performed in the healthy control group. Children with values below the laboratory reference range for any of these parameters were not included in the growing pains group. The exact number of children excluded due to low ferritin, 25-hydroxyvitamin D, vitamin B12, or folate levels was not systematically recorded. Children with identified deficiencies received appropriate clinical evaluation, treatment, and follow-up in pediatric outpatient clinics as part of routine clinical care.

### 2.2. Sample Size Calculation

An a priori sample size calculation was performed using G*Power version 3.1.9.4, based on the planned comparison of CSHQ total scores between the growing-pains and healthy-control groups. With a medium effect size (Cohen’s d = 0.50), a two-sided alpha level of 0.05, and 80% power, the required sample was 128 participants, corresponding to a minimum of 64 children per group. The final analysis included 138 participants.

### 2.3. Questionnaire Administration

All assessments were performed during the outpatient clinic visit after the diagnosis of growing pains had been established by a board-certified pediatric rheumatologist according to the Peterson diagnostic criteria. All study assessments were coordinated by a developmental pediatrician using standardized procedures. Pain assessments were obtained directly from the children using age-appropriate instruments, whereas the sleep and maternal anxiety questionnaires were completed independently by the mothers. All questionnaires were reviewed for completeness and scored by the developmental pediatrician before statistical analysis without influencing participants’ responses.

### 2.4. Outcome Measures

#### 2.4.1. Pain Severity

Pain severity was evaluated with validated self-report instruments selected according to the child’s age. Children were asked to rate the intensity of pain they generally experienced when growing-pain episodes occurred, rather than pain at the time of the clinic visit or during a single specified episode.

For children younger than 8 years, pain severity was measured using the Faces Pain Scale–Revised (FPS-R) [13]. The FPS-R consists of six facial expressions representing increasing pain intensity, scored as 0, 2, 4, 6, 8, or 10. Children were asked to select the face that best represented the intensity of pain they generally experienced during growing-pain episodes.

Children aged 8 years or older rated pain severity on a 10 cm horizontal Visual Analog Scale (VAS), with endpoints labeled “no pain” and “worst possible pain” [14]. Children were asked to mark the point on the line that best represented the intensity of pain they generally experienced during growing-pain episodes. The distance from the “no pain” anchor to the marked point was measured with a ruler and expressed as a score from 0 to 10.

Scores derived from the two instruments were not mathematically combined. For each child, a single pain-intensity score obtained using the age-appropriate instrument was expressed on a common 0–10 metric. No additional mathematical conversion or rescaling was therefore performed, and this score was used as a continuous pain-severity variable in the statistical analyses.

#### 2.4.2. Sleep Habits

Sleep habits were assessed with the Children’s Sleep Habits Questionnaire (CSHQ), a parent-reported instrument designed to characterize sleep-related behaviors in children [15]. The 33-item CSHQ assesses eight dimensions of childhood sleep: bedtime resistance, sleep onset delay, sleep duration, sleep anxiety, night wakings, parasomnias, sleep-disordered breathing, and daytime sleepiness. Items were scored according to their designated response format, with reverse scoring used when required. The CSHQ total score was obtained by summing the item scores, with higher values reflecting greater sleep disturbance. A total score > 41 was considered above the established CSHQ screening cut-off; however, this threshold is not diagnostic of a specific sleep disorder [15,16]. The Turkish adaptation of the CSHQ has been shown to have satisfactory validity and reliability [16]. Although the original CSHQ was developed and validated primarily for children aged 4–10 years, subsequent cross-cultural validation studies have evaluated adapted versions of the CSHQ in samples including children up to 12 years of age [17,18].

#### 2.4.3. Maternal Anxiety

Maternal anxiety was evaluated with the Generalized Anxiety Disorder-7 (GAD-7), a seven-item self-report questionnaire that measures anxiety symptoms experienced over the preceding two weeks [19]. Each item is scored from 0 (“not at all”) to 3 (“nearly every day”), producing a total score ranging from 0 to 21, with higher scores indicating greater anxiety symptom severity. The Turkish adaptation of the GAD-7 has demonstrated satisfactory validity and reliability [20].

### 2.5. Statistical Analysis

All statistical analyses were conducted using SPSS version 15.0 (SPSS Inc., Chicago, IL, USA). Categorical data are reported as *n* (%), whereas continuous data are summarized as mean ± standard deviation or median (minimum–maximum), depending on distributional characteristics. Normality of continuous variables was evaluated using the Shapiro–Wilk test together with inspection of skewness and kurtosis values. A Shapiro–Wilk *p* value ≥ 0.05 was taken as evidence against a statistically significant departure from normality. Because formal normality tests may be sensitive to minor distributional deviations, variables with skewness and kurtosis values between −1 and +1 were additionally regarded as approximately normally distributed. The choice between parametric and nonparametric methods was based on these distributional assessments. Continuous variables were compared between groups using the independent-samples *t* test or Mann–Whitney U test according to distributional characteristics. Categorical comparisons were made with Pearson’s chi-square test, and associations between continuous variables were examined using Spearman’s rank correlation.

For Mann–Whitney U analyses, effect size was calculated as r = |z|/√N, where N represents the total number of observations in both groups. Between-group differences were further summarized using the Hodges–Lehmann estimator with 95% CI. For 2 × 2 categorical analyses, Cramér’s V and, where applicable, the odds ratio with 95% CI were reported.

Multiple linear regression was used to examine independent associations while adjusting for potential confounders. In the overall study population, the CSHQ total score was entered as the dependent variable, and study group (growing pains vs. control), age, sex, daily screen time, maternal educational level, number of siblings, and maternal GAD-7 score were included as independent variables. Because CSHQ total scores were not normally distributed in the overall sample, a log10 transformation was applied before regression analysis.

A separate multiple linear regression model was fitted within the growing-pains group to identify factors independently associated with CSHQ total score. Pain severity, maternal GAD-7 score, age, sex, daily screen time, and maternal educational level were entered as predictors. Because CSHQ total scores were approximately normally distributed in this subgroup, no transformation was applied.

Maternal educational level was treated as a categorical variable, with university education or higher as the reference category and dummy variables created for high school and primary/middle school education. Continuous independent variables were entered in their original form. Potential multicollinearity was assessed using tolerance and variance inflation factor (VIF) values. Sensitivity analyses were repeated in the subgroup aged 4–10 years, which corresponds to the validated age range of the Turkish CSHQ. A two-sided *p* value below 0.05 was taken to indicate statistical significance.

## 3. Results

### 3.1. Participant Characteristics

A total of 138 children aged 4–12 years were analyzed, comprising 71 with growing pains and 67 healthy controls. Mean age was 7.49 ± 2.68 years, and 80 participants (58.0%) were girls.

No significant group differences were found for age, sex, body weight, body mass index, household smoking exposure, or weekly physical activity (all *p* > 0.05). Significant differences were observed for height (*p* = 0.037), number of siblings (*p* = 0.023), daily screen time (*p* = 0.001), and maternal educational level (*p* < 0.001) (Table 1).

### 3.2. Sleep Habits and Maternal Anxiety

The median CSHQ total score was significantly higher in children with growing pains than in healthy controls [49.0 (33.0–81.0) vs. 42.0 (31.0–79.0), respectively; *p* < 0.001], with a Mann–Whitney effect size of *r* = 0.320. The Hodges–Lehmann estimate of the between-group difference (growing pains − control) was 6 points (95% CI 3–9) (Table 2). By contrast, maternal GAD-7 scores were comparable between the two groups [3.0 (0–18.0) vs. 2.0 (0–13.0), respectively; *p* = 0.531] (Table 2).

When participants were categorized according to the established CSHQ screening cut-off, scores > 41 were more frequent among children with growing pains than among healthy controls [54/71 (76.1%) vs. 35/67 (52.2%), respectively; χ^2^ = 8.539, *p* = 0.003]. The association corresponded to a Cramér’s V of 0.249, and the odds of scoring above the screening cut-off were higher in the growing-pains group than in controls (OR = 2.91, 95% CI 1.40–5.99) (Table 3).

### 3.3. Pain Severity and Its Associations with Sleep and Maternal Anxiety

In the growing-pains group, pain severity ranged from 0.3 to 10.0, with a mean of 5.9 ± 2.53 and a median of 6.0. A positive correlation was observed between pain severity and CSHQ total scores (*r* = 0.355, *p* = 0.002). No statistically significant correlation was observed between pain severity and maternal GAD-7 scores (*r* = 0.026, *p* = 0.829). Likewise, maternal GAD-7 and CSHQ total scores were not significantly correlated (*r* = 0.201, *p* = 0.093) (Table 4).

### 3.4. Multivariable Analyses

In the overall study population, the multiple linear regression model was statistically significant (F [8, 129] = 4.176, *p* < 0.001), accounting for 20.6% of the variance in log10-transformed CSHQ total scores (adjusted R^2^ = 0.156). After adjustment for age, sex, daily screen time, maternal educational level, number of siblings, and maternal GAD-7 score, belonging to the growing pains group remained independently associated with higher log10-transformed CSHQ total scores (B = 0.039, standardized β = 0.203, 95% CI: 0.006–0.072, *p* = 0.020). Higher maternal GAD-7 scores were also independently associated with higher log10-transformed CSHQ total scores (B = 0.007, standardized β = 0.239, 95% CI: 0.002–0.011, *p* = 0.003). Compared with maternal university education or higher, primary/middle-school maternal education was independently associated with higher log10-transformed CSHQ total scores (B = 0.054, standardized β = 0.246, 95% CI: 0.009–0.100, *p* = 0.019). Age, sex, daily screen time, high-school maternal education, and number of siblings were not significantly associated with CSHQ total scores (Table 5).

Among children with growing pains, the multiple linear regression model was statistically significant (F [7, 63] = 2.223, *p* = 0.044) and explained 19.8% of the variance in CSHQ total scores (adjusted R^2^ = 0.109). Greater pain severity was independently associated with higher CSHQ total scores (B = 1.144, standardized β = 0.260, 95% CI: 0.096–2.191, *p* = 0.033). Maternal GAD-7 score, age, sex, daily screen time, and maternal educational level were not significantly associated with CSHQ total scores (Table 5).

### 3.5. Sensitivity Analysis

In the sensitivity analysis restricted to children aged 4–10 years (*n* = 109; 53 with growing pains and 56 healthy controls), the principal findings remained consistent with those of the primary analysis. The median CSHQ total score remained significantly higher in children with growing pains than in healthy controls [50.0 (35.0–81.0) vs. 42.0 (31.0–71.0), respectively; *p* < 0.001], with an effect size of r = 0.446 and a Hodges–Lehmann estimate of 9 points (95% CI 5–12). CSHQ scores above the screening cut-off were also more frequent in the growing-pains group than in controls [43/53 (81.1%) vs. 29/56 (51.7%), respectively; *p* = 0.001]. Within the growing pains subgroup, pain severity remained positively correlated with CSHQ total scores (r = 0.332, *p* = 0.015). Detailed results are presented in Supplementary Table S1.

## 4. Discussion

In this comparative cross-sectional study, children with growing pains had significantly higher CSHQ total scores and were more likely to have scores above the established screening cut-off than healthy controls. Importantly, the association between growing pains and higher CSHQ scores remained significant after adjustment for age, sex, daily screen time, maternal educational level, number of siblings, and maternal GAD-7 score. In the overall study population, higher maternal GAD-7 scores were also independently associated with higher CSHQ total scores. Among children with growing pains, greater pain severity was independently associated with higher CSHQ total scores, whereas maternal GAD-7 scores were not significantly associated with sleep disturbance. Maternal anxiety levels also did not differ significantly between the growing pains and control groups and were not associated with pain severity. Taken together, these findings suggest that sleep disturbance is an important component of the clinical burden of growing pains. The association with maternal anxiety differed according to the analytic population: maternal GAD-7 scores were associated with sleep disturbance in the overall sample, but this association was not statistically significant within the growing-pains group.

Sleep disturbance appears to be an important component of the clinical burden associated with growing pains. In a recent Danish cohort study, Hestbæk et al. reported disturbed sleep in approximately one quarter of children with growing pains [21]. Similarly, Zhang et al. found that 43.3% of children with growing pains experienced difficulty sleeping and 43.0% reported nocturnal awakenings during pain episodes [1]. More recently, Guo et al. reported several sleep-related characteristics associated with growing pains, including fear of sleeping alone, prolonged sleep latency, and nocturnal awakenings. Notably, a sleep latency of 30–60 min remained independently associated with growing pains after multivariable adjustment [22]. Earlier polysomnographic evidence demonstrated a significantly higher frequency of periodic limb movements of sleep in children with growing pains, with the association remaining significant after adjustment for potential confounders [23]. Our findings are consistent with these observations and extend previous evidence by demonstrating higher CSHQ total scores and a greater proportion of scores above the established screening cut-off in children with growing pains than in healthy controls using a validated multidimensional sleep questionnaire. Notably, more than half of the healthy controls also had CSHQ scores above the screening cut-off. Because the CSHQ is a screening instrument rather than a diagnostic tool, scores above this threshold should not be interpreted as indicating a specific sleep disorder. This finding may also reflect the relatively high sensitivity of the questionnaire to common sleep-related behaviors in otherwise healthy children. Importantly, growing pains remained significantly associated with higher CSHQ scores after controlling for age, sex, daily screen time, maternal educational level, number of siblings, and maternal GAD-7 score. These findings suggest that sleep disturbance may constitute a clinically relevant aspect of growing pains rather than simply reflecting between-group differences in demographic or lifestyle factors. Furthermore, the positive correlation between pain severity and CSHQ total scores indicates that greater pain severity is accompanied by greater sleep disturbance. However, because of the cross-sectional design of the present study, the temporal direction of this association cannot be determined. The use of a multidimensional sleep measure may be particularly relevant in this context, as a recent scoping review of 61 studies found that outcome assessment in growing pains has largely focused on pain-related measures, with relatively limited evaluation of broader psychosocial and functional dimensions [5].

An additional consideration is the clinical overlap between growing pains and restless legs syndrome (RLS). Both conditions may present with evening or nocturnal lower-extremity symptoms and sleep disturbance, which can complicate clinical differentiation. A recent narrative review by Rosen et al. highlighted that, although growing pains and RLS are generally considered distinct conditions, they share several clinical and biological features, including nocturnal leg symptoms, sleep disturbance, familial aggregation, iron deficiency, and low vitamin D levels [24]. Recent literature has also emphasized the broader neuropsychiatric context of pediatric RLS. A recent review highlighted associations between pediatric RLS and several psychiatric and neurodevelopmental conditions, particularly attention-deficit/hyperactivity disorder, while anxiety and depressive symptoms may also coexist and complicate the clinical presentation [25]. Similarly, a recent scoping review by Smith et al. identified substantial heterogeneity in the assessments and diagnostic criteria used for growing pains and persistent lower-limb pain in the presence of RLS, emphasizing the lack of consistent diagnostic approaches and the need for more standardized assessment practices [5]. Because the four essential RLS criteria and family history of RLS were not assessed systematically in all participants in the present study, some degree of diagnostic overlap cannot be excluded and should be considered when interpreting the sleep-related findings.

Parental psychological factors have increasingly been recognized as relevant to pediatric pain. Psychosocial factors remain relatively understudied in growing pains. A recent systematic review and meta-analysis comprising 37 studies and 16,086 participants found that only two studies specifically examined psychosocial factors, with results that were not fully consistent [3]. Few studies have directly evaluated parental psychological status in the context of growing pains. Uziel et al., in a study including parents of 37 children with growing pains and 21 matched healthy controls, found similar anxiety scores between the groups, although maternal depression was significantly higher among mothers of children with growing pains [2]. Beveridge et al., drawing on 49 studies in a systematic review and meta-analysis, reported associations between parental anxiety and both pediatric chronic pain and poorer pain-related functioning in children [9]. Similarly, Martí et al. found associations between parental psychological factors, including anxiety and depressive symptoms, and several pain-related outcomes in children and adolescents with chronic pain [7]. However, the authors also emphasized that much of the available evidence was derived from cross-sectional studies with moderate or high risk of bias, limiting causal interpretation. Regarding the direct between-group comparison, maternal GAD-7 scores did not differ significantly between mothers of children with growing pains and those of healthy controls. This finding is consistent with the limited direct evidence available in growing pains.

In the separate multivariable analysis of the overall study population, higher maternal GAD-7 scores were independently associated with higher CSHQ total scores after adjustment. However, when the multivariable regression analysis was restricted to the growing-pains group, the association between maternal GAD-7 and CSHQ total scores was not statistically significant after adjustment for pain severity, age, sex, daily screen time, and maternal educational level. Maternal GAD-7 scores were also not significantly associated with pain severity within this subgroup.

The different patterns of association between maternal anxiety and sleep disturbance in the overall sample and the growing-pains subgroup may reflect the interplay of general family-related factors and condition-specific symptoms. Maternal anxiety may be a marker of broader family-level psychological distress, potentially influencing bedtime routines, emotional interactions, or maternal perceptions of children’s sleep difficulties. Previous studies have emphasized the relevance of parental psychological functioning and family-related factors in pediatric chronic pain [7,9]. In contrast, among children with growing pains, recurrent evening or nocturnal pain may be more immediately relevant to sleep initiation and continuity. Pain-related discomfort may therefore emerge as a more prominent correlate of sleep disturbance within this clinical subgroup, whereas maternal anxiety may remain one of several background psychosocial influences. This interpretation is consistent with the independent association between pain severity and CSHQ scores observed in our growing-pains subgroup and with previous evidence describing the complex, bidirectional relationship between pain and sleep in children [4]. However, these explanations remain hypothetical. Although pain severity was independently associated with sleep disturbance in children with growing pains, maternal anxiety may still contribute to sleep-related difficulties in this population. The relative contributions of these factors cannot be established from the present cross-sectional findings.

Methodological considerations may also help explain the observed pattern. The smaller sample size in the growing-pains subgroup may have reduced statistical power to detect an independent association between maternal anxiety and sleep disturbance, particularly given the number of predictors included in the subgroup model. Furthermore, the two regression models differed in their covariates and outcome transformations, limiting direct comparisons of their coefficients. Importantly, an association reaching statistical significance in the overall sample but not in the subgroup does not necessarily indicate that the underlying associations are significantly different [26]. Thus, the nonsignificant association within the growing-pains subgroup should not be interpreted as evidence that maternal anxiety is unrelated to sleep disturbance. Larger longitudinal studies incorporating repeated assessments of pain severity, sleep patterns, maternal psychological distress, and family functioning are needed to better understand these potentially overlapping relationships.

Recent evidence from pediatric clinical populations further supports the complex interplay among sleep disturbance, affective symptoms, pain, and disease-related factors. In pediatric inflammatory bowel disease, Ballesio et al. reported prospective associations between baseline inflammatory biomarkers and subsequent anxiety and insomnia symptoms; specifically, the CRP-to-albumin ratio predicted anxiety symptoms, whereas albumin was associated with both anxiety and insomnia symptoms at one-year follow-up [27]. Szabo et al., using repeated daily assessments and actigraphy in youth with inflammatory bowel disease, found that longer sleep onset latency was associated with pain on the following day, whereas no significant associations were observed between sleep parameters and negative affect [28]. In adolescents with chronic pain, Palermo et al. showed that insomnia symptoms remained elevated over 12 months and that chronic pain, poorer sleep hygiene, and greater depressive symptoms were associated with a more persistent course of insomnia [29]. Evidence from pediatric autoinflammatory disease also indicates that sleep disturbances may occur outside periods of overt disease activity. Taşkın et al. observed frequent sleep disturbances in clinically stable, attack-free children with familial Mediterranean fever (FMF) who had normal inflammatory markers; however, sleep scores showed no significant differences by age, sex, or MEFV exon 10 mutation burden [30]. Similarly, Baris Cetinkaya and Battal reported a modest association between poorer sleep quality and greater depressive symptom severity in clinically stable pediatric FMF, whereas disease severity was not significantly associated with sleep quality or depressive symptoms [31]. Taken together, these findings support a multifactorial model of sleep disturbance in pediatric clinical populations, in which pain, emotional factors, and clinical or biological characteristics may contribute simultaneously. This broader context is consistent with our finding that maternal GAD-7 scores were associated with sleep disturbance in the overall sample, while pain severity remained the significant correlate within the growing-pains group.

The association between greater pain severity and higher CSHQ scores in our study is consistent with evidence that sleep and pain may interact bidirectionally in pediatric populations. Importantly, this association remained significant in the multivariable analysis after adjustment for maternal GAD-7 score, age, sex, daily screen time, and maternal educational level, suggesting that the observed relationship was not fully explained by these measured factors. Oh et al. described a cyclical relationship between sleep and pain in children and adolescents with chronic pain and noted that sleep loss may intensify the pain experience [4]. Similarly, a review of the pediatric literature by Morris et al. suggested that poor sleep, particularly reduced sleep efficiency and nighttime awakenings, may predict subsequent pain, whereas evidence for the reverse direction—from pain to later sleep disturbance—was less consistent [32]. Longitudinal evidence further supports this relationship. Liu et al. found that frequent pain predicted incident insomnia symptoms one year later, while baseline insomnia symptoms also predicted subsequent frequent pain in adolescents [33]. More recently, Roman-Juan et al. reported that greater pain interference predicted subsequent sleep disturbance over a 12-month period in children and adolescents with chronic pain, with fatigue contributing to this longitudinal association [34]. Although growing pains differ from the chronic pain conditions represented in these studies, their characteristic evening and nocturnal occurrence makes sleep particularly relevant to the clinical experience. Accordingly, the association observed in our study may reflect the impact of recurrent nocturnal pain on sleep, an influence of disturbed sleep on the pain experience, or a reciprocal relationship between the two. However, because our study was cross-sectional, the temporal direction of these pathways could not be determined.

Daily screen time was significantly higher in children with growing pains than in healthy controls in the unadjusted comparison. Previous systematic reviews have reported associations between greater electronic media exposure and poorer sleep outcomes in children and adolescents [35]. However, in our study, daily screen time was not independently associated with CSHQ total scores in the multivariable models, suggesting that the between-group difference in screen exposure did not independently account for the observed difference in sleep disturbance. Lower parental socioeconomic indicators, including lower parental educational level, have also been associated with greater sleep disturbance in pediatric populations [36]. In our study, primary/middle-school maternal education was associated with higher CSHQ scores in the overall population compared with university education or higher, whereas maternal educational level was not significantly associated with CSHQ scores within the growing pains group. The lack of statistical significance within the growing-pains subgroup may reflect the smaller sample size and reduced statistical power of the subgroup analysis, potentially limiting the ability to detect an association observed in the overall population. These findings should therefore be interpreted cautiously and may reflect broader socioeconomic, environmental, or family-related influences on sleep that were not directly assessed.

This study has several strengths. Growing pains were diagnosed according to established diagnostic criteria by a pediatric rheumatologist, and the study included an age- and sex-comparable healthy control group. Pain severity was assessed directly from the children using age-appropriate self-report instruments, while sleep habits and maternal anxiety were evaluated using validated questionnaires. In addition, the simultaneous assessment of pain severity, sleep habits, and maternal anxiety allowed these interrelated dimensions to be examined within the same cohort. Furthermore, multivariable analyses allowed the associations of growing pains and pain severity with sleep disturbance to be examined after accounting for several potentially relevant demographic, behavioral, and maternal psychological factors.

Several limitations should also be acknowledged. Importantly, the four essential diagnostic criteria for RLS and family history of RLS were not assessed systematically in all participants. Therefore, possible overlap between growing pains and RLS could not be reliably excluded, which should be considered when interpreting the sleep-related findings. In addition, the single-center setting and relatively small sample size may restrict the generalizability of the findings. Furthermore, the cross-sectional design precludes conclusions regarding the temporal or causal relationship between pain and sleep disturbance.

The exact numbers of potentially eligible control-group children whose families declined participation and children excluded from the growing-pains group because of low ferritin, 25-hydroxyvitamin D, vitamin B12, or folate levels were not systematically recorded. Consequently, definitive recruitment, participation, and screening exclusion rates could not be calculated. This limitation may have introduced selection bias and should be considered when interpreting the findings and their generalizability.

Another methodological limitation concerns pain assessment. Pain severity was assessed retrospectively based on children’s recall of their usual pain intensity during growing-pain episodes rather than through real-time pain diaries or ecological momentary assessment, which may have introduced recall bias. Additionally, although the FPS-R and VAS were selected according to age and expressed on a common 0–10 scale, treating scores from these two distinct instruments as a single continuous variable may have introduced minor measurement variability.

Sleep habits were assessed by maternal report and maternal anxiety by a self-report questionnaire, which may have introduced reporting bias. Previous evidence suggests that parental psychological factors may influence the perception and reporting of children’s symptoms [10]. Additionally, objective sleep assessments, including actigraphy and polysomnography, were not available. Although the study included children aged 4–12 years, the Turkish version of the CSHQ was validated in children aged 4–10 years. However, a sensitivity analysis restricted to the validated age range yielded findings consistent with the primary analysis, supporting the robustness of the main results. Individual CSHQ subscales were also not analyzed because the prespecified primary sleep outcome was the total CSHQ score; therefore, potentially relevant differences across specific sleep domains could not be evaluated. Future longitudinal studies should examine individual CSHQ subscales to identify which specific sleep domains are most disrupted in children with growing pains. Finally, although several potential confounders were included in the multivariable analyses, residual confounding cannot be excluded, and broader socioeconomic, environmental, and family-level factors were not directly assessed.

## 5. Conclusions

Children with growing pains exhibited greater sleep disturbance than healthy controls and were more likely to have CSHQ scores above the established screening cut-off. The association between growing pains and higher CSHQ scores remained significant after adjustment for relevant demographic, behavioral and maternal psychological factors. In the overall study population, higher maternal GAD-7 scores were independently associated with greater sleep disturbance, whereas this association was not statistically significant within the growing-pains group. Among children with growing pains, greater pain severity was independently associated with greater sleep disturbance, whereas maternal GAD-7 scores were not significantly associated with either sleep disturbance or pain severity within this subgroup. These findings highlight sleep as an important dimension of the clinical burden of growing pains and support the consideration of sleep assessment in affected children, particularly in those with greater pain severity. Further longitudinal and interventional research is needed to clarify the temporal sequence between pain and sleep disturbance and to evaluate whether sleep-targeted interventions can improve clinical outcomes in children with growing pains.

## Figures and Tables

**Table 1:** Demographic and Clinical Characteristics of the Study Groups.

Variable	Growing Pains (*n* = 71)	Control (*n* = 67)	*p*
Age, years	7.0 (4.0–12.0)	8.0 (4.0–12.0)	0.720
Male sex, *n* (%)	31 (43.7)	27 (40.3)	0.689
Body weight, kg	23.0 (12.0–65.0)	25.0 (15.0–90.0)	0.163
Height, cm	121.0 (98.0–165.0)	128.0 (106.0–182.0)	0.037
Body mass index, kg/m^2^	15.7 (10.9–25.6)	15.06 (11.1–27.1)	0.453
Number of siblings	1.64 ± 1.24	1.23 ± 0.81	0.023
Household smoking exposure, *n* (%)	35 (49.3)	34 (50.7)	0.865
Daily screen time, h	3.0 (0–9.0)	2.0 (0.5–5.0)	0.001
Physical activity, days/week	4.0 (0–7.0)	4.0 (0–7.0)	0.402
Maternal educational level, *n* (%)			<0.001
Primary/middle school	28 (39.4)	7 (10.4)	
High school	22 (31.0)	25 (37.3)	
University or higher	21 (29.6)	35 (52.2)	

Data are presented as median (minimum–maximum), mean ± standard deviation, or *n* (%), as appropriate. Number of siblings was normally distributed and is presented as mean ± standard deviation; the other continuous variables are presented as median (minimum–maximum).

**Table 2:** Comparison of Sleep Habits and Maternal Anxiety Scores Between the Study Groups.

Variable	Growing Pains (*n* = 71)	Control (*n* = 67)	*p*	Effect Size, *r*	Hodges–Lehmann Estimate (95% CI)
CSHQ total score	49.0 (33.0–81.0)	42.0 (31.0–79.0)	<0.001	0.320	6 (3–9)
GAD-7 score	3.0 (0–18.0)	2.0 (0–13.0)	0.531	0.062	0 (−1 to 0)

Data are presented as median (minimum–maximum). Effect size *r* was calculated for Mann–Whitney U comparisons as |z|/√N. Hodges–Lehmann estimates are presented as growing pains − control. CSHQ, Children’s Sleep Habits Questionnaire; GAD-7, Generalized Anxiety Disorder-7; CI, confidence interval.

**Table 3:** Distribution of CSHQ Scores According to the Screening Cut-off by Study Group.

CSHQ Classification	Growing Pains (*n* = 71)	Control (*n* = 67)	Total (*n* = 138)	χ^2^	*p*	Cramér’s V	OR (95% CI)
CSHQ ≤ 41	17 (23.9%)	32 (47.8%)	49 (35.5%)				
CSHQ > 41	54 (76.1%)	35 (52.2%)	89 (64.5%)	8.539	0.003	0.249	2.91 (1.40–5.99)
Total	71 (100%)	67 (100%)	138 (100%)				

Data are presented as n (% within each study group). The odds ratio is presented for CSHQ > 41 in the growing-pains group relative to the control group. CSHQ, Children’s Sleep Habits Questionnaire; OR, odds ratio; CI, confidence interval.

**Table 4:** Correlations between pain severity, sleep habits, and maternal anxiety in children with growing pains.

Variables	Spearman’s *r*	*p* Value
Pain severity–CSHQ total score	0.355	0.002
Pain severity–Maternal GAD-7 score	0.026	0.829
CSHQ total score–Maternal GAD-7 score	0.201	0.093

Abbreviations: CSHQ, Children’s Sleep Habits Questionnaire; GAD-7, Generalized Anxiety Disorder-7. Spearman’s rank correlation analysis was used. A *p* value < 0.05 was considered statistically significant.

**Table 5:** Multiple Linear Regression Analyses of Factors Associated With CSHQ Total Scores.

**A. Overall study population (*n* = 138)**				
Predictor	B	Standardized β	95% CI for B	*p*
Growing pains group vs. control	0.039	0.203	0.006 to 0.072	0.020
Age, years	−0.005	−0.137	−0.011 to 0.001	0.111
Male sex	0.010	0.050	−0.022 to 0.041	0.542
Daily screen time, h	0.005	0.090	−0.005 to 0.016	0.318
Maternal education: high school	0.025	0.121	−0.012 to 0.061	0.189
Maternal education: primary/middle school	0.054	0.246	0.009 to 0.100	0.019
Number of siblings	−0.006	−0.066	−0.022 to 0.010	0.457
Maternal GAD-7 score	0.007	0.239	0.002 to 0.011	0.003
**B. Children with growing pains (*n* = 71)**				
Predictor	B	Standardized β	95% CI for B	*p*
Pain severity	1.144	0.260	0.096 to 2.191	0.033
Maternal GAD-7 score	0.483	0.162	−0.201 to 1.168	0.163
Age, years	−0.830	−0.213	−1.801 to 0.140	0.092
Male sex	2.026	0.091	−3.214 to 7.267	0.443
Daily screen time, h	−0.121	−0.019	−1.633 to 1.390	0.873
Maternal education: high school	−0.425	−0.018	−7.087 to 6.237	0.899
Maternal education: primary/middle school	5.045	0.222	−1.516 to 11.606	0.129

Model statistics: (A) R^2^ = 0.206; adjusted R^2^ = 0.156; F(8,129) = 4.176; *p* < 0.001. (B) R^2^ = 0.198; adjusted R^2^ = 0.109; F(7,63) = 2.223; *p* = 0.044. B, unstandardized regression coefficient; β, standardized regression coefficient; CI, confidence interval; CSHQ, Children’s Sleep Habits Questionnaire; GAD-7, Generalized Anxiety Disorder-7. In Panel A, the dependent variable was the log10-transformed CSHQ total score; in Panel B, the untransformed CSHQ total score was used. Control group, female sex, and maternal university education or higher were the reference categories.

## Data Availability

The datasets generated and/or analyzed during the current study are available from the corresponding author upon reasonable request.

## Supplementary Materials

The following supporting information can be downloaded at: https://www.mdpi.com/article/10.3390/children13101429/s1, Table S1: Sensitivity Analysis Restricted to Children Aged 4–10 Years.
