# Supplementary material for: Sleep Habits and Maternal Anxiety in Children with Growing Pains: A Comparative Cross-Sectional Study
Source: Children (Basel). 2026 Oct 18;13(10):1429. doi: 10.3390/children13101429 (PMC13702860; doi:10.3390/children13101429)
Supplement: Supplementary file 1 [file children-13-01429-s001.zip › children-4554973-supplementary.pdf]

Supplementary Table S1. Sensitivity Analysis Restricted to Children Aged 4–10 Years

**Outcome**	**Growing pains (n = 53)**	**Healthy controls (n = 56)**	**Effect estimate**	***p* value**
**CSHQ total score**	50.0 (35.0–81.0)	42.0 (31.0–71.0)	*r* = 0.446; Hodges–Lehmann estimate= 9 (95% CI 5–12)	**<0.001**
**Maternal GAD-7 score**	3.0 (0–18.0)	2.0 (0–13.0)	*r* = 0.091; Hodges–Lehmann estimate = 0 (95% CI 0–2)	0.344
**CSHQ >41, n (%)**	43/53 (81.1%)	29/56 (51.7%)	Cramér's V = 0.310; OR = 4.00 (95% CI 1.70–11.11)	**0.001**

Within the growing pains subgroup

**Association**	**Spearman's *r***	***p* value**
**Pain severity – CSHQ total score**	0.332	**0.015**
**Pain severity – Maternal GAD-7 score**	−0.018	0.896
**CSHQ total score – Maternal GAD-7 score**	0.165	0.238

Footnote: Data are presented as median (minimum–maximum) or n (%), as appropriate. Effect sizes for Mann–Whitney U comparisons are reported as r. Hodges–Lehmann estimates represent the between-group location shift calculated as growing pains minus healthy controls. OR for CSHQ >41 represents the odds of a score above the screening cut-off in children with growing pains relative to healthy controls. CSHQ, Children's Sleep Habits Questionnaire; GAD-7, Generalized Anxiety Disorder-7; CI, confidence interval; OR, odds ratio.
